# Automatic Detection and Quantification of Acute Cerebral Infarct by Fuzzy Clustering and Histographic Characterization on Diffusion Weighted MR Imaging and Apparent Diffusion Coefficient Map

**DOI:** 10.1155/2014/963032

**Published:** 2014-03-12

**Authors:** Jang-Zern Tsai, Syu-Jyun Peng, Yu-Wei Chen, Kuo-Wei Wang, Hsiao-Kuang Wu, Yun-Yu Lin, Ying-Ying Lee, Chi-Jen Chen, Huey-Juan Lin, Eric Edward Smith, Poh-Shiow Yeh, Yue-Loong Hsin

**Affiliations:** ^1^Department of Electrical Engineering, National Central University, Jhongli City, Taoyuan County 32001, Taiwan; ^2^Department of Computer Science and Information Engineering, National Central University, Jhongli City, Taoyuan County 32001, Taiwan; ^3^Department of Neurology, Landseed Hospital, Pingzhen City, Taoyuan County 32449, Taiwan; ^4^Department of Neurology, National Taiwan University Hospital, Taipei City 10002, Taiwan; ^5^Department of Medical Imaging, Landseed Hospital, Pingzhen City, Taoyuan County 32449, Taiwan; ^6^Department of Radiology, Taipei Medical University-Shuang Ho Hospital, New Taipei City 23561, Taiwan; ^7^Department of Neurology, Chi-Mei Medical Center, Tainan City 71004, Taiwan; ^8^Department of Clinical Neurosciences and Hotchkiss Brain Institute, University of Calgary, Calgary, AB, Canada T2N 1N4; ^9^Epilepsy Center, Buddhist Tzu Chi General Hospital, Hualian City, Hualian County 97002, Taiwan; ^10^Biomedical Electronics Translational Research Center, National Chiao Tung University, Hsinchu City 30010, Taiwan; ^11^Department of Neurology, Chung Shan Medical University Hospital, Taichung City 40201, Taiwan

## Abstract

Determination of the volumes of acute cerebral infarct in the magnetic resonance imaging harbors prognostic values. However, semiautomatic method of segmentation is time-consuming and with high interrater variability. Using diffusion weighted imaging and apparent diffusion coefficient map from patients with acute infarction in 10 days, we aimed to develop a fully automatic algorithm to measure infarct volume. It includes an unsupervised classification with fuzzy C-means clustering determination of the histographic distribution, defining self-adjusted intensity thresholds. The proposed method attained high agreement with the semiautomatic method, with similarity index 89.9 ± 6.5%, in detecting cerebral infarct lesions from 22 acute stroke patients. We demonstrated the accuracy of the proposed computer-assisted prompt segmentation method, which appeared promising to replace the laborious, time-consuming, and operator-dependent semiautomatic segmentation.

## 1. Introduction

Cerebrovascular disease is one of the leading causes of acute mortality and chronic disability [1]. The volume of infarct is associated with severity of acute ischemic stroke and correlates with clinical prognosis and the effect of endovascular therapy [2–4]. A rapid and reliable method of determination of volume of acute infarct will help predict the prognosis and facilitate further investigation.

The diffusion weighted imaging (DWI) is more sensitive than other magnetic resonance imaging (MRI) modalities to small water diffusion changes in the acute ischemic brain, especially within 48 hours of the ictus [5–9].

Automatic algorithms for segmentation for acute infarct in MRI have been reported [10–15]. The unsupervised method developed by Li et al. was based on a multistage procedure including image preprocessing, calculation of tensor field, measurement of diffusion anisotropy, segmentation of infarct volume based on adaptive multiscale statistical classification, and partial volume voxel reclassification [11]. Bhanu Prakash et al. used a probabilistic neural network for selecting infarct slices and an adaptive Gaussian mixture model for segmentation of the infarcts [12]. Hevia-Montiel et al. developed a method for cerebral infarct lesion segmentation from DWI by applying nonparametric density estimation [13]. Gupta et al. identified the infarct slices and the hemisphere automatically in DWI based on the difference in the percentile characteristics of intensity normalized images and parameters of infarct slice identification and infarct hemisphere identification [14]. Shen et al. detected infarct lesions based on the voxel intensity segmentation and the spatial location of tissue distribution [15].

We aimed to design a DWI-based computer-assisted method to provide clinicians a prompt and accurate determination of the volumes of acute cerebral infarct. The operation of this method is based on the histographic characteristic of the output clusters of a fuzzy C-means (FCM) clustering [16]. Additional measures were taken to ensure the accuracy of infarct detection, including discriminating infarcts from artifacts due to magnetic inhomogeneity by incorporating the histographic information in the apparent diffusion coefficient (ADC) map.

## 2. Materials and Methods

### 2.1. Subjects and Image Acquisition

Landseed Hospital has been participating in the nationwide Taiwan Stroke Registry, which prospectively registered patients with stroke onset within 10 days according to a preestablished system [17]. For this study, we recruited 22 patients (11 women and 11 men, 62–83 years of age) with acute cerebral infarction and MRI examinations during January-February 2011. The protocol of this research has been reviewed and approved by the Institutional Review Board (IRB) of Landseed Hospital.

All MRIs were acquired with a Signa HDxt 1.5T Optima edition (GE Healthcare, Waukesha, WI) and consisted of a DWI scan (TR/TE/Flip angle = 6000 ms/82.8 ms/90°, FOV = 230 × 230 mm^2^, matrix = 128 × 128, in-plane resolution = 1.79 × 1.79 mm, 24 axial slices, 5 mm slice thickness with 1 mm gap) and an ADC map with *b* = 1000 s/mm^2^.

### 2.2. Automatic Infarct Detection Procedure

**Figure 1:**
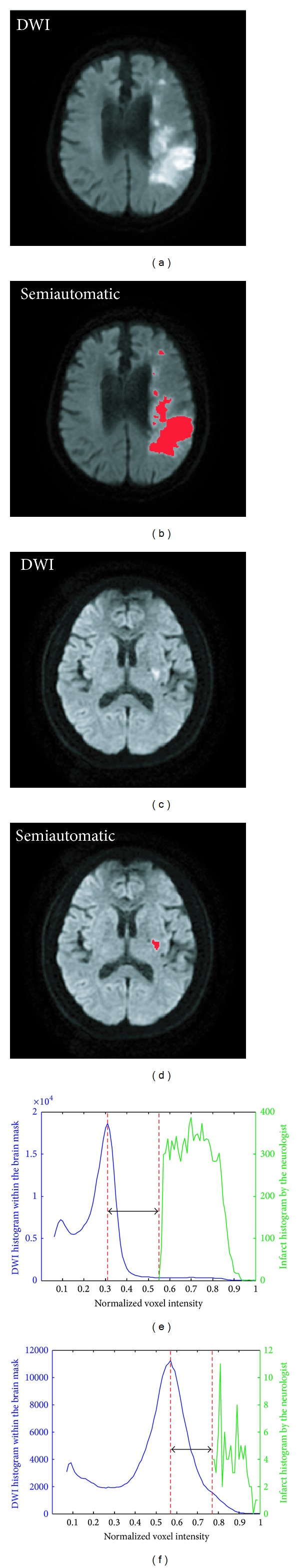
The histographic characteristics of the raw DWI and the infarct. (a) The raw DWI of patient number 20. (b) The semiautomatically demarcated infarcts, printed in red, of patient number 20. The infarct volume = 46.828 mL. The lower bound of the infarcts = *I*_peak_ + 0.24. (c) The raw DWI of patient number 3. (d) The semiautomatically demarcated infarcts, printed in red, of patient number 3. The infarct volume = 0.399 mL. The lower bound of the infarcts = *I*_peak_ + 0.21. (e) The histogram of the normalized voxel intensity within the brain mask of the whole-brain DWI and the intensity distribution of the semiautomatically demarcated infarcts of patient number 20. (f) The histogram of the normalized voxel intensity within the brain mask of the whole-brain DWI and the intensity distribution of the semiautomatically demarcated infarcts of patient number 3.

The proposed method was developed on a personal computer with Intel Core i5 CPU, 2.67 GHz processor speed, and 4 GB RAM. The infarct detection procedure was carried out mainly with a MATLAB program (The MathWorks, Inc., Natick, MA). We utilized the histographic characteristic of the DWI for infarct detection.  Figure 1 illustrates one example with a large infarct volume and another with a small infarct volume. The procedure comprised the following steps.

**Step 1 (coregistration and intensity normalization)**

The ADC map was registered to the corresponding DWI by a rigid registration (translation and rotation) and a trilinear interpolation based on the normalized mutual information method to correct for differences due to head movements [18]. The DWI and registered ADC map were normalized so that their intensities were both distributed in a standardized range (0,1). The program we used to run this step was the Statistical Parametric Mapping 8 (SPM8, Wellcome Department of Cognitive Neurology, London, UK).

**Step 2 (extracting the brain mask from the whole-brain DWI)**

The brain mask was extracted from the whole-brain DWI based on the estimation of the inner and outer skull surfaces by using BET (Brain Extraction Tool), a software package developed at FMRIB Centre, University of Oxford, Oxford, United Kingdom [19]. Note that the fractional intensity threshold was set at 0.3, smaller than the default value 0.5, to give a larger brain outline estimate, which would completely enclose the brain. On one hand, the brain mask extraction would not eliminate any portion of the cerebral infarcts. On the other hand, no part of the brain skull portion enclosed by the brain outline would be mistaken as an infarct region in the subsequence steps, because there are obvious intensity differences between the cerebral infarcts and brain skull.

**Step 3 (preclustering elimination)**

The histogram of DWI within the brain mask was smoothed by a third-order moving-average filter. The peak of the smoothed histogram was identified. The normalized intensity, denoted by *I*_peak_, corresponding to this histographic peak was used as a threshold. The voxels with normalized intensities lower than or equal to *I*_peak_ would be eliminated from further processing.

**Step 4 (FCM clustering)**

The remaining voxels after the previous step were divided into 50 clusters by an unsupervised classification with the conventional FCM clustering algorithm.

**Step 5 (skimming the clusters for candidate voxels)**

The clusters with mean normalized intensity larger than the normalized intensity of the histographic peak plus 0.2, that is, *I*_peak_ + 0.2, were selected. The voxels belonging to these selected clusters would be treated as candidate voxels of infarct in the next step.

**Step 6 (eliminating labels with insufficient intensity)**

Each cluster of candidate voxels of infarct was further divided into one or several labels, at most the same number as the voxel number in that cluster. A label comprised connected voxels. The labels with average normalized intensity lower than or equal to the threshold (*I*_peak_ + 0.2) were eliminated from further processing.

**Step 7 (eliminating labels with weak edge)**

The labels with weak edge were eliminated from further processing. The edge map was extracted from normalized DWI by using Canny edge detector [20]. The low and high threshold values were defined as (0,0.3). The parameter of the standard deviation of the Gaussian filter was determined to be 1.

**Step 8 (eliminating candidate labels due to magnetic inhomogeneity)**

Let *I*_peak,ADC_ denote the intensity corresponding to the histographic peak of the ADC map. Further, let *I*_lower  mean,ADC_ denote the average intensity of the lower-intensity half of all the voxels in a specific label on the ADC map. For this specific label, if the ratio *I*_lower mean,ADC_/*I*_peak,ADC_ ≥ 0.5, this label was considered an artifact caused by magnetic inhomogeneity. All artifacts thus defined were detected and eliminated in this step. Finally, all the voxels in the remaining labels in the remaining clusters were taken to be infarct.

### 2.3. Performance Evaluation

A voxelwise comparison between the proposed automatic segmentation and semiautomatic segmentation by the experienced neurologist (Chen) [21] gives the four parameters of each patient: true positive (TP), true negative (TN), false positive (FP), and false negative (FN). The sensitivity (Sen.), specificity (Spe.), positive prediction value (PPV), and negative prediction value (NPV) are calculated from the four parameters [22].

The similarity index (SI) is used to indicate the degree of agreement between the infarcts detected by our method and those detected semiautomatically by the neurologist. Its formula is SI = 2 × TP/(2 × TP + FP + FN) [23]. In addition, Cohen's kappa coefficient is also calculated. It eliminates the agreement due to random chance and is considered a conservative measure of interrater agreement. Infarct volume was calculated as the summation of the detected infarct area of axial DWIs times the slice thickness [24]. The agreement evaluation of volume measurements of the proposed algorithm was carried out by calculating the intraclass correlation coefficient (ICC) [25].

### 2.4. Preliminary Experiment

A preliminary experiment was conducted to find the most suitable cluster numbers for the FCM clustering in Step 4. Cluster numbers ranging from 6 to 100 were tested. For each of the tested cluster numbers, a semiprocedure of the proposed method was conducted on each of the 22 recruited patients to obtain an average SI. The semiprocedure consisted of Steps E1 to E7, whereof Steps E1 to E5 were the same as Steps 1 to 5. Steps E6 to E7 were as follows.

*Step E6 (selecting infarct labels)*. Each cluster of candidate voxels of infarct was further divided into one or several labels, at most the same number as the voxel number in that cluster. A label comprised connected voxels. All the labels containing at least one voxel belonging to a semiautomatically demarcated infarct region were selected as infarct labels.

*Step E7 (SI calculation)*. The SI was calculated with the voxel-by-voxel comparison between the infarct labels selected in Step 6 and the semiautomatically demarcated infarct regions.

## 3. Results

**Figure 2:**
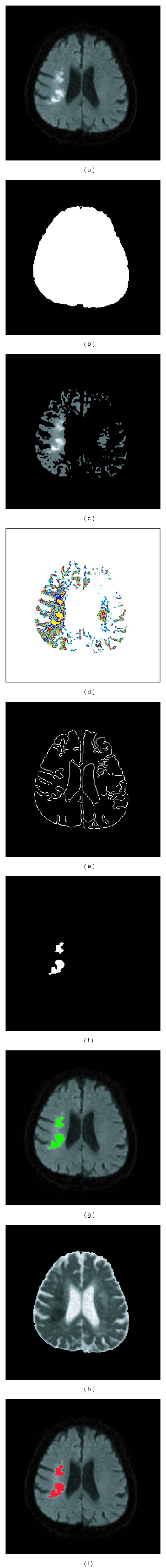
Illustrating the procedure of the proposed method with exemplary images. (a) The raw DWI. (b) The brain mask. (c) The region-of-interest map derived after the preclustering elimination in Step 3. (d) The FCM cluster map for the 50 clusters in Step 4. (e) Canny edge detection map. (f) The computer-assisted infarct segmentation result. (g) The detected infarct was mapped to raw DWI. (h) The ADC map. (i) The semiautomatically demarcated infarct regions by the experienced neurologist.

The exemplary images in Figure 2 illustrate the procedure of the proposed method.  Figure 2(a) shows an example of the DWI slice used as the input to the proposed method in Step 1.  Figure 2(b) was a slice of the whole-brain mask extracted from the whole-brain DWI in Step 2.  Figure 2(c) shows a slice of the output of the preclustering elimination in Step 3. These were the DWI voxels with normalized intensities higher than *I*_peak_ within the brain mask. In Figure 2(d), different colors were used to paint the voxels of different clusters in an exemplary DWI slice after the FCM clustering in Step 4.  Figure 2(e) shows the corresponding Canny edge detection map.  Figure 2(f) shows an example of the final detected infarcts after further processing through Steps 5, 6, 7, and 8.  Figure 2(g) is a combination of the detected infarcts and the raw DWI.  Figure 2(h) shows the ADC map that was used in Step 8 to eliminate artifact-induced spurious infarcts.  Figure 2(i) shows the result of the semiautomatic infarct segmentation by the neurologist on the same input DWI.

**Figure 3:**
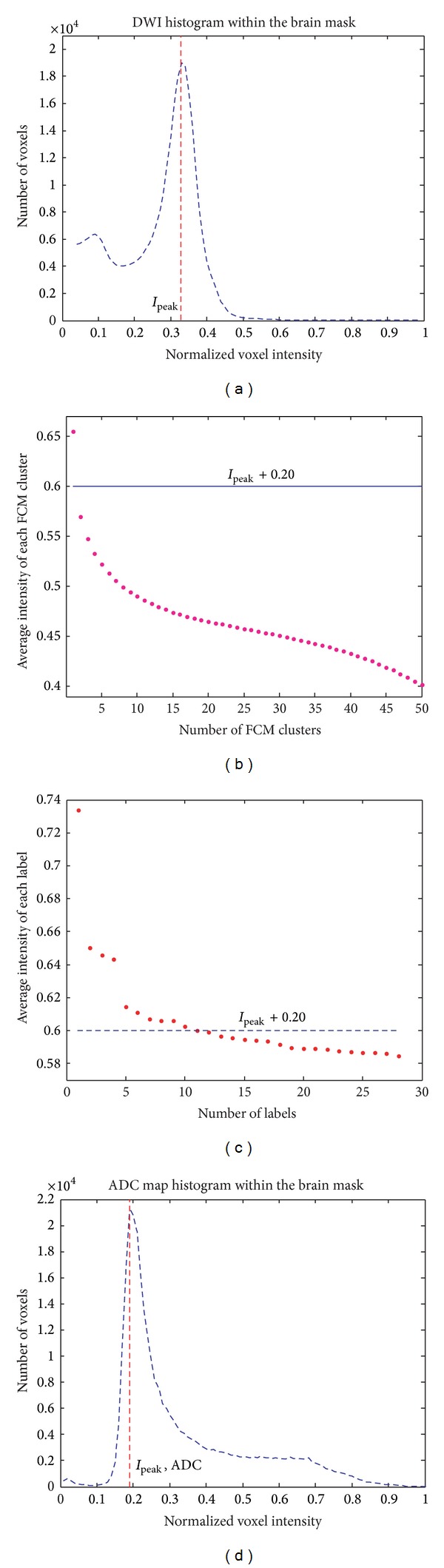
(a) The histogram of the normalized voxel intensity within the brain mask of a whole-brain DWI. (b) The average normalized intensities of the 50 clusters created in Step 4. (c) The average normalized intensities of the labels from the clusters of candidate voxels of infarct in Step 5. (d) The histogram of the normalized voxel intensity within the brain mask of an ADC map.

Figure 3 shows how the histographic information of the voxel intensity was utilized in the proposed method to facilitate the identification of infarct. The blue curve in Figure 3(a) represents the smoothed histogram of the voxel intensity of a normalized raw DWI. The intensity corresponding to the peak of the histogram is referred to as *I*_peak_. In Step 3, all voxels with normalized intensity lower than or equal to *I*_peak_ were eliminated because they very unlikely belonged to infarct areas. This preclustering elimination greatly reduced the computation load in the latter steps. Each of the 50 dots in Figure 3(b) represents the average normalized intensity of all the voxels in an individual cluster among the 50 output clusters of the FCM clustering in Step 4. In Step 5, only the clusters with mean normalized intensity higher than or equal to *I*_peak_ + 0.2 were skimmed (selected) as candidate infarct clusters. In the example shown in Figure 3(b), only one cluster became a candidate cluster. Each dot in Figure 3(c) represents the average normalized intensity of an individual label in the candidate cluster(s). The labels with average normalized intensities lower than *I*_peak_ + 0.2 were eliminated in Step 6.  Figure 3(d) shows the histogram of the normalized voxel intensity of the ADC map. It was used in Step 8 to distinguish artifacts due to magnetic inhomogeneity.

Verified with the reference by the experienced neurologist (Chen), our algorithm had high sensitivity, specificity, and SI.  Table 1 tabulates the performance indices of our algorithm on each of the 22 subjects. Note that we conducted the proposed method 10 times on each patient's MRI to get a (mean ± standard deviation), shown in the supplementary table (see Supplementary Material available online at http://dx.doi.org/10.1155/2014/963032), of each performance index.  Table 1 only shows the mean values of the performance indices. With total infarct lesion volume ranging from 0.155 to 482.939 mL, the sensitivity was 88.036 ± 12.117%, the specificity 99.992 ± 0.024%, and the SI 89.933 ± 6.460%. The standard deviations shown in the supplementary table reveal that the variation due to FCM clustering was low and acceptable.

**Figure 4:**
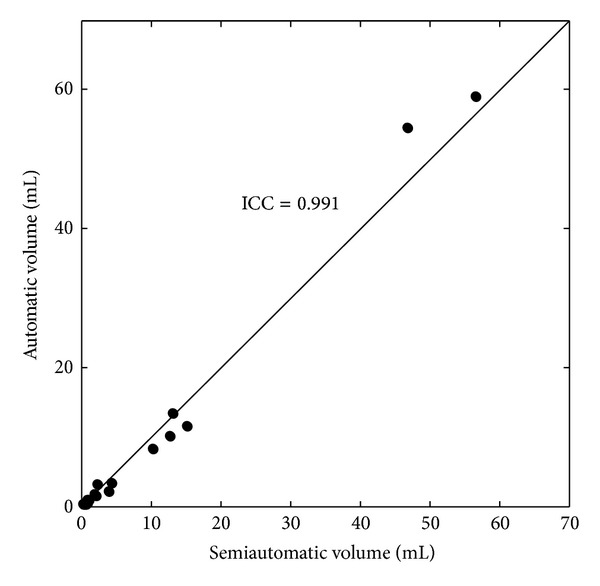
Automatically segmented versus semiautomatically segmented infarct volumes.

Figure 4 shows the relationship in the infarct volumes obtained by the semiautomatic method and the proposed method. The infarct volumes determined by the proposed method correlated well with those determined by the semiautomatic method with an ICC of 0.991. Notice that patient number 22 was an outlier, with a much higher infarct volume than those of the other patients. This patient is not included in the plot of Figure 4. The ICC with patient number 22 included was 0.993.

**Figure 5:**
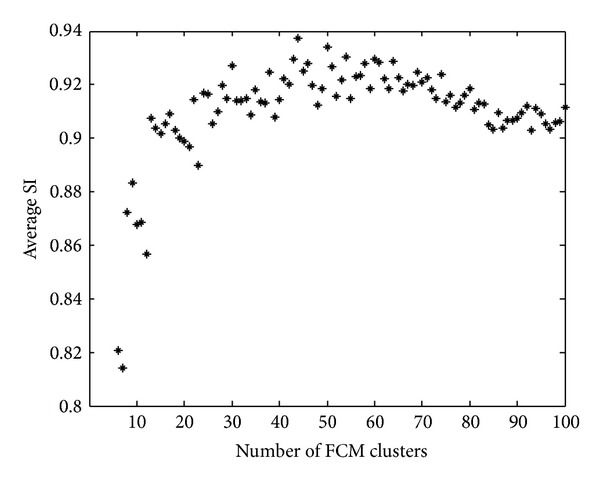
The result of the preliminary experiment to determine the FCM cluster number. Each of the average SI values was the average SI of the semiprocedure of the proposed method conducted on the 22 patients.

**Figure 6:**
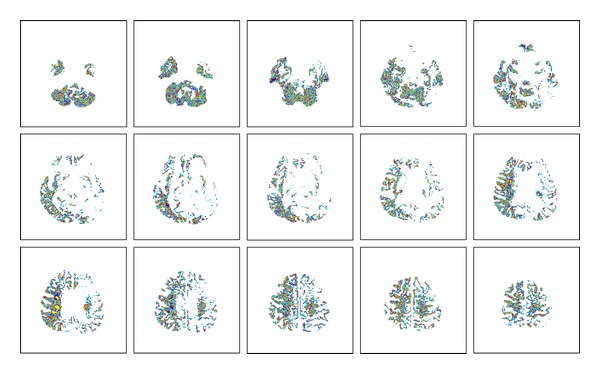
Different colors represent the voxels of the 50 different clusters in a whole-brain DWI of patient number 9 in Step 4.

The result of the preliminary experiment with various output cluster numbers of the FCM clustering algorithm is shown in Figure 5. It demonstrates that the number of the output clusters has substantial influence on the average SI value.  Figure 6 illustrates the result of the FCM clustering by using different colors to represent the 50 different clusters in a whole-brain DWI.

**Figure 7:**
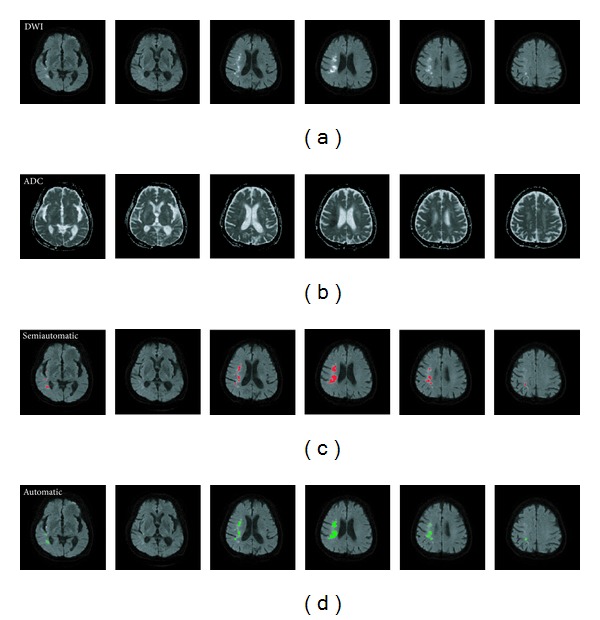
Illustrating the input and output images of the proposed algorithm using patient number 9 as an example. This figure shows 6 of the 23 slices of the whole-brain MRIs of patient 9. (a) Six axial slices of DWI. (b) Six axial slices of ADC map. (c) Infarct regions, painted red, semiautomatically demarcated by the neurologist. (d) Infarct regions, painted green, detected automatically by the proposed algorithm.

To illustrate the effect of the proposed algorithm, the semiautomatic and the proposed automatic segmentation results on patient #9 are displayed in Figure 7.

It took our personal computer less than 90 seconds to execute from Step 1 through Step 8. However, because the SPM program in Step 1 and the BET program in Step 2 required user intervention, the whole procedure actually took nearly 5 minutes. In the future, if the functions currently executed by the SPM and BET programs are integrated into the main MATLAB program, the whole procedure will finish within 90 seconds.

## 4. Discussion

We reported a high SI of our algorithm. The method proposed by Bhanu Prakash et al. attained 60% in SI [12]. The SI of their proposed method two years later [10] was improved to 67%. Another method [14] proposed by the same team attained 67.6% in SI. The method [15] by Shen et al. could attain 87.9% in the average SI, obtained with simulated lesions. The method [11] proposed by Li et al. attained SI above 92%. In comparison, the performance of our method in terms of SI is higher than those of Prakash and Gupta's team, similar to the method of Shen et al., and only next to that of the method of Li et al. It is worth noting that the SI values reported by different research teams were based on different gold standards and calculated from different experiment setups. Hence, there is no fair comparison among the performances of these methods.

We attribute the high SI of our method to the following key points.

First, we used a self-adaptive threshold, that is, *I*_peak_, in the preclustering elimination step (Step 3). The purpose of the preclustering elimination was to increase the efficiency of fuzzy clustering and reduce the computation time. The histographic peak corresponded to the most abundant intensity of the DWI image. The voxels with intensity lower than *I*_peak_ were very unlikely to belong to an infarct, so it is safe to eliminate them from further processing. The threshold was self-adaptable in that the *I*_peak_ of every patient served the purpose well.

Second, we used an optimal value for the output cluster number of the FCM clustering algorithm in Step 4. It was chosen to be 50 since this was a value that would lead to high SI values, as was demonstrated in Figure 5.

**Figure 8:**
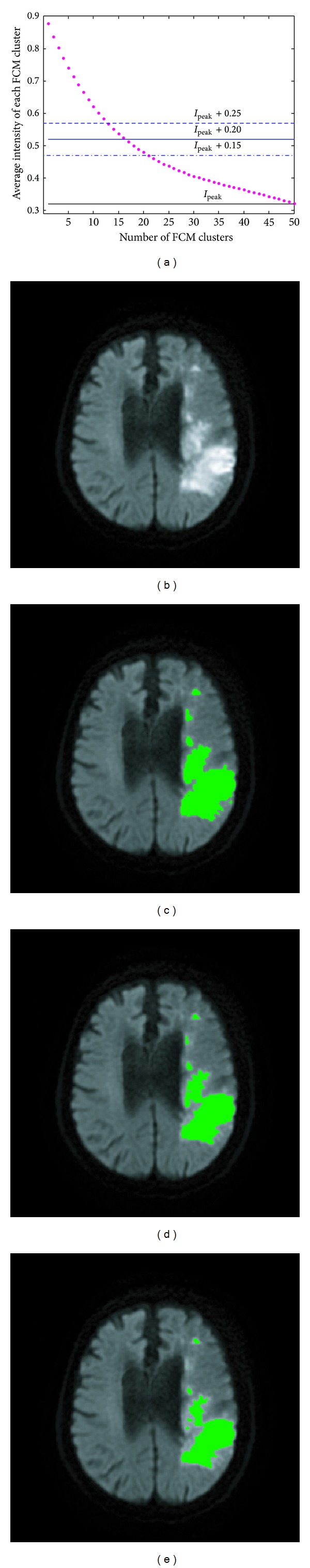
The effect of threshold variation of clusters skimming in Step 5. (a) Each red dot represents the average normalized intensity of an individual cluster among the 50 output clusters of the FCM clustering. (b) The raw DWI. (c) The detected infarct lesions with skimming from *I*_peak_ + 0.15. (d) The detected infarct lesions with skimming from *I*_peak_ + 0.2. (e) The detected infarct lesions with skimming from *I*_peak_ + 0.25. The SI values of the three cases were 88.4%, 93.3%, and 99.2%, whereas the sensitivities were 100.0%, 99.9%, and 98.4%, respectively.

Third, in Step 5, we selected the mean demarcation threshold, *I*_peak_ + 0.2, used by the neurologist as the threshold for skimming the clusters for candidate voxels. As illustrated in Figures 8(c), 8(d), and 8(e), different extents of skimming led to different SI values. We found that the difference between *I*_peak_ and the minimum normalized intensity of the infarct regions demarcated semiautomatically by the neurologist averaged around 0.2. In the proposed algorithm, the normalized intensity value that was 0.2 higher than *I*_peak_ was selected for the value from which the skimming started. Note that the demarcation threshold *I*_peak_ + 0.2 had been determined based on the statistics of the experienced neurologist's semiautomatic demarcation results. The level of this threshold can be changed to accommodate for different neurologists, scanners, and acquisition parameters. A suitable new threshold can be obtained by statistical analysis on a training set, making the proposed algorithm adoptable to all situations.

**Figure 9:**
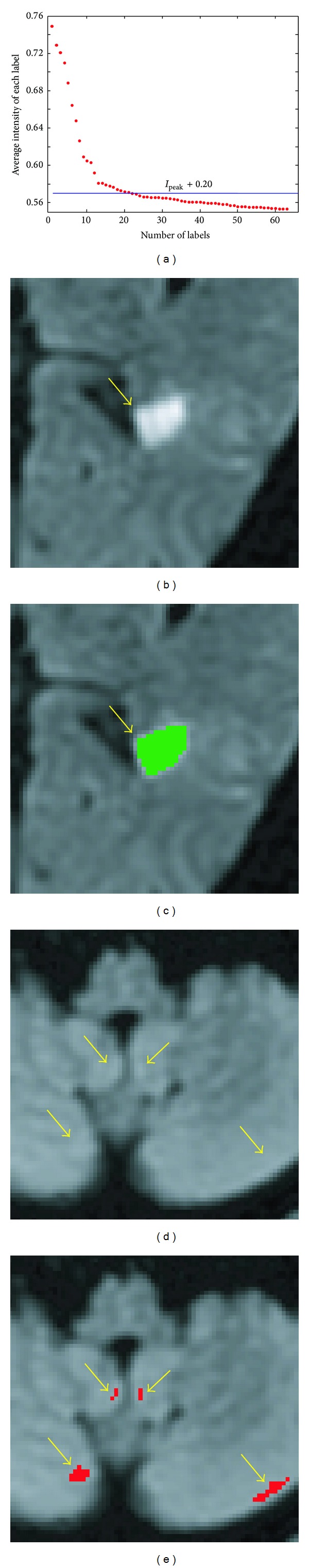
In Step 6, labels in the skimmed cluster(s) would be eliminated if their individual average normalized intensities were lower than or equal to the threshold level, namely, *I*_peak_ + 0.2. (a) The red dots represent the average normalized intensities of the labels in the cluster(s) that had been skimmed in Step 5. The blue line indicates the threshold level in this step. (b) A typical label, pointed to by the arrow, with its average normalized intensity higher than the threshold level would appear white possibly with a faint suburb. (c) Such a label, painted green, was classified as an infarct region. (d) The arrows point to labels with individual average normalized intensities lower than the threshold level. They appeared faint all over. (e) Such labels, painted red, were classified as noninfarct regions.

Fourth, we eliminated false-positive labels in the candidate clusters. The results before adopting Step 6 had shown that there would be some false-positive infarct regions in the result if all the voxels of the skimmed clusters were taken as infarct. In Step 6, by dividing each skimmed cluster into labels and eliminating labels with low intensity, the false positives could mostly become true negatives. As illustrated in Figure 9(a), the labels with average normalized intensity higher than or equal to *I*_peak_ + 0.2 were retained in the candidates and the others were eliminated.  Figure 9(b) exemplifies a true-positive infarct label, which looked white and might have a faint suburb. Such a label was classified as an infarct region, painted green in Figure 9(c).  Figure 9(d) shows some labels with average normalized intensity lower than *I*_peak_ + 0.2; these labels looked faint all over. Such labels were classified as noninfarct regions, painted red in Figure 9(e).

**Figure 10:**
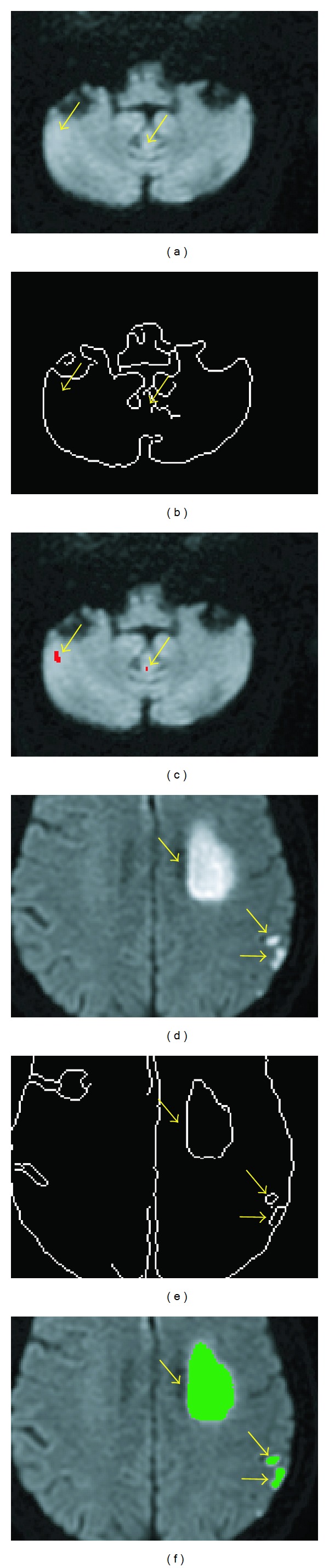
In Step 7, labels selected in Step 6 would be eliminated if their edges were weak. (a)-(b) The labels pointed to by the arrows were all labels that had been selected in Step 6 for further processing. (c) In the Canny edge detection map, the arrow-pointed-to labels in (a) did not have corresponding edges because their edges were weak. (d) In the Canny edge detection map, the arrow-pointed-to labels in (b) had corresponding edges because their edges were not weak. (e) The arrow-pointed-to labels in (b) were classified as noninfarct regions, painted red. (f) The arrow-pointed-to labels in (b) were classified as infarct regions, painted green.

Fifth, edge detection was used to further eliminate labels with weaker edges. The results of Step 6 still contained a few false-positive labels. That is to say, some noninfarct labels had sufficient normalized intensity to pass the decisions in Steps 5 and 6. However, these false-positive labels had weaker edges than real infarct labels had.  Step 7 used this edge for eliminating the false-positive labels.  Figure 10 demonstrates the function of this step with real examples.

**Figure 11:**
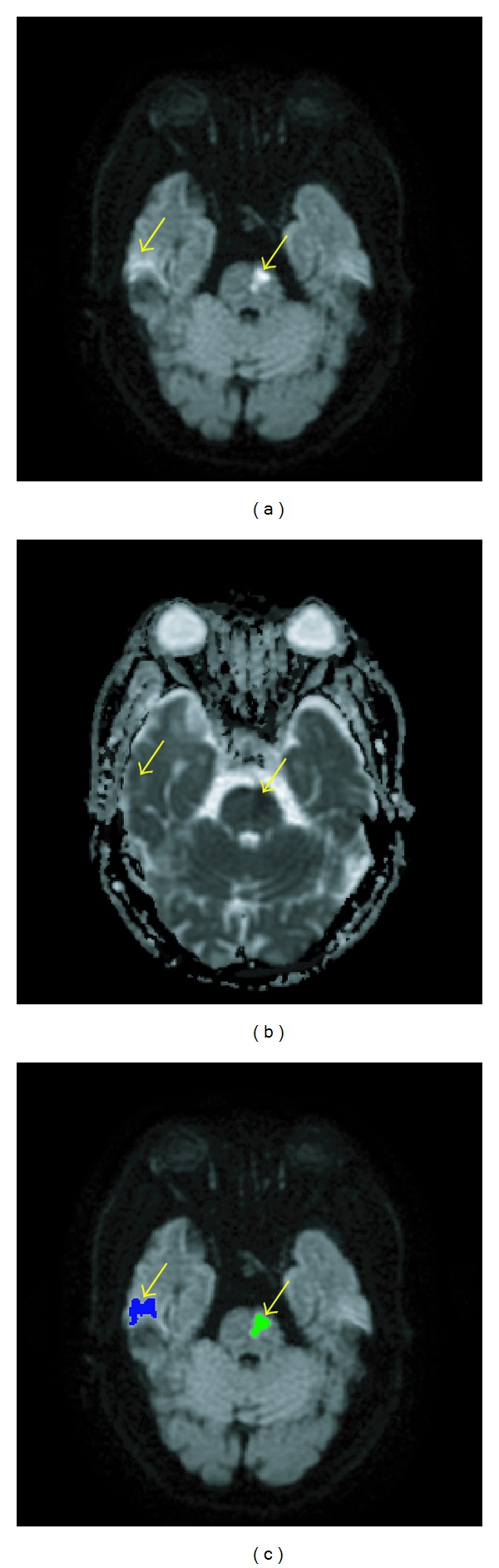
In Step 8, candidate labels due to magnetic inhomogeneity were eliminated. (a) In this example, the two labels pointed by arrows had intensities higher than *I*_peak_ + 0.2 and appeared equally bright in the DWI. (b) The two labels had different intensities in the ADC map. (c) The artifact (blue) was differentiated from the infarct (green) in Step 8.

Sixth, we detected and eliminated the false-positive labels due to magnetic-inhomogeneity artifacts. The result of Step 7 still contained false-positive labels, which were actually artifacts caused by the magnetic susceptibility differences between adjacent air and cerebrospinal fluid structures and the surrounding soft tissues with echo-planar imaging techniques [26]. In the DWI, the magnetic inhomogeneity created artifacts with intensities commensurate with those of infarcts. It had been a difficult task to eliminate the artifacts [12]. However, in the ADC map, the artifact intensity was higher than the infarct intensity. This property was used in Step 8 to detect and eliminate the artifacts caused by magnetic inhomogeneity.  Figure 11 illustrates this phenomenon.

The proposed method had the second lowest SI (79.722%), despite of 100.000% sensitivity, for patient number 13 among the 22 patients. That was actually due to inconsistent selection of threshold by the neurologist. Notice from Table 1 that the demarcation boundary selected by the neurologist for patient number 13 was *I*_peak_ + 0.31, which was much higher than the average value. In other words, the neurologist included fewer voxels into infarct than usual. The demarcation boundary selected by the neurologist for patient number 14 was *I*_peak_ + 0.14, much lower than the average value. That is to say, more voxels than usual were included into the infarct by the neurologist. That was why the proposed method had the lowest SI (74.359%) and the lowest sensitivity (59.444%) for patient number 14 among all the patients. These cases demonstrated the inconsistency in the semiautomatic way of segmentation. In contrast, the proposed method attained consistent segmentation, because it used the same value as the threshold in all cases.

Although the performance of the proposed method has been satisfactory, an even higher SI is still desirable. At present, the magnetic inhomogeneity does pose a limitation in further increasing the accuracy of infarct detection. To attain higher SI, it will be necessary to find a smarter way than the method we use in Step 8 to identify artifacts due to magnetic inhomogeneity.

This proposed infarct detection method will also be useful for the development of the automatic detection of white matter lesions. It can increase the accuracy of white matter lesion detection by excluding infarct lesions, which could be mistook for white matter lesions easily.

## 5. Conclusion

This proposed algorithm for acute infarct segmentation provides a prompt calculation of acute infarct volume from the DWI and ADC map. The infarct detection was achieved with fuzzy clustering that divided the DWI into ordered clusters based on voxel intensities. Under careful scrutiny on the intensity spectrum, candidate clusters were skimmed and deceptive labels further eliminated. Additionally, the ADC map was used to help identify spurious infarcts that were actually artifacts caused by magnetic inhomogeneity. This method attained high similarity indices. With the semiautomatic segmentation by the experienced neurologist for comparison, this automatic method attained high similarity indices.

Our future research will emphasize finding a more effective way to deal with the magnetic inhomogeneity to attain a higher accuracy in infarct segmentation. Applying the proposed infarct detection method to facilitate accurate computer-assisted segmentation of white matter lesions will also be our future effort.

## Supplementary Material

Supplementary Table: The statistics of computer-assisted segmentation of cerebral infarct of individual patient after repeating the proposed method for 10 times. The values of mean±standard deviation of each statistical method for individual patient were shown to demonstrate the consistence of results of our proposed algorithm.

## Figures and Tables

**Table 1:** Demographics and the statistics of the computer-assisted cerebral infarct segmentation results of individual patients after repeating the proposed method for 10 times.

Patient	Sex	Age	Demar.	Total infarct volume (mL)		ΔVol.	SI (%)	Kappa (%)	Sen. (%)	Spe. (%)	PPV (%)	NPV (%)
				Semi-auto.	Auto.							
1	M	73	0.27	0.155	0.212	36.6%	84.661	84.661	100.000	99.999	73.529	100.000
2	M	80	0.19	0.358	0.377	5.3%	97.438	97.438	100.000	100.000	95.014	100.000
3	F	69	0.21	0.399	0.381	−4.4%	97.716	97.716	95.556	100.000	100.000	100.000
4	F	84	0.22	0.474	0.378	−20.3%	86.835	86.834	78.037	100.000	98.349	99.998
5	F	70	0.2	0.501	0.337	−32.7%	91.662	91.662	93.398	100.000	91.723	100.000
6	M	63	0.19	0.545	0.451	−17.2%	90.413	90.412	82.764	100.000	100.000	99.999
7	M	55	0.2	0.612	0.471	−22.9%	82.684	82.683	73.190	100.000	95.072	99.998
8	F	66	0.15	0.644	0.479	−25.6%	85.332	85.331	74.436	100.000	100.000	99.998
9	M	61	0.21	0.796	0.895	12.5%	94.143	94.142	100.000	99.999	88.938	100.000
10	M	64	0.19	1.003	1.019	1.6%	98.927	98.927	99.710	100.000	98.171	100.000
11	F	75	0.25	1.675	1.794	7.1%	94.373	94.372	97.619	99.998	91.568	99.999
12	F	56	0.19	1.966	1.497	−23.9%	86.438	86.434	76.140	100.000	100.000	99.992
13	F	74	0.31	2.141	3.232	51.0%	79.722	79.715	100.000	99.984	66.312	100.000
14	F	86	0.14	3.749	2.228	−40.6%	74.359	74.349	59.444	100.000	100.000	99.977
15	M	76	0.19	4.143	3.448	−16.8%	85.827	85.819	78.898	99.997	95.789	99.987
16	F	87	0.17	10.108	8.351	−17.4%	90.453	90.440	82.621	100.000	100.000	99.975
17	F	83	0.16	12.657	10.088	−20.3%	88.603	88.582	79.662	100.000	99.952	99.958
18	F	57	0.19	13.063	13.492	3.3%	97.656	97.652	99.254	99.992	96.124	99.999
19	M	72	0.19	15.014	11.611	−22.7%	87.181	87.159	77.332	100.000	99.992	99.955
20	M	80	0.24	46.828	54.497	16.4%	92.426	92.374	99.990	99.894	85.933	100.000
21	M	74	0.22	56.517	59.147	4.7%	97.526	97.503	99.793	99.956	95.365	99.998
22	M	91	0.1	482.939	429.534	−11.1%	94.147	93.675	88.942	100.000	100.000	99.056

Mean		72.5	0.20			−6.2%	89.933	89.904	88.036	99.992	94.174	99.949
STDEV		10.4	0.04			22.3%	6.460	6.446	12.117	0.024	8.886	0.200

Note: Demar.: the lowest intensity, above *I*_peak_, of the infarcts demarcated by the neurologist; SI: similarity index; Sen.: sensitivity; Spe.: specificity; PPV: positive predictive value; NPV: negative predictive value; STDEV: standard deviation; ΔVol. = (Semi-auto. vol. − Auto. vol.)/Semi-auto. vol.
