# Supplementary material for: Automatic Detection and Quantification of Acute Cerebral Infarct by Fuzzy Clustering and Histographic Characterization on Diffusion Weighted MR Imaging and Apparent Diffusion Coefficient Map
Source: Biomed Res Int. 2014 Mar 12;2014:963032. doi: 10.1155/2014/963032 (PMC3971548; doi:10.1155/2014/963032)
Supplement: Supplementary file 1 — Supplementary Table: The statistics of computer-assisted segmentation of cerebral infarct of individual patient after repeating the proposed method for 10 times. The values of mean±standard deviation of each statistical method for individual patient were shown to demonstrate the consistence of results of our proposed algorithm. [file 963032.f1.pdf]

Supplementary: The statistics of the computer-assisted infarct segmentation results of individual patients after repeating the proposed method for 10 times

Patient	Total infarct volume (ml)		SI (%)	kappa (%)	Sen. (%)	Spe. (%)	PPV (%)	NPV (%)
	Semi-auto	Auto						
1	0.155	0.212±0.014	84.661±3.301	84.661±3.301	100.000±0.000	99.999±0.000	73.529±4.937	100.000±0.000
2	0.358	0.377±0.006	97.438±0.769	97.438±0.769	100.000±0.000	100.000±0.000	95.014±1.465	100.000±0.000
3	0.399	0.381±0.009	97.716±1.124	97.716±1.124	95.556±2.160	100.000±0.000	100.000±0.000	100.000±0.000
4	0.474	0.378±0.041	86.835±2.509	86.834±2.509	78.037±4.498	100.000±0.000	98.349±5.222	99.998±0.000
5	0.501	0.337±0.026	91.662±1.726	91.662±1.726	93.398±1.384	100.000±0.000	91.723±3.838	100.000±0.000
6	0.545	0.451±0.039	90.413±4.429	90.412±4.429	82.764±7.188	100.000±0.000	100.000±0.000	99.999±0.001
7	0.612	0.471±0.017	82.684±1.146	82.683±1.146	73.190±0.273	100.000±0.000	95.072±3.243	99.998±0.000
8	0.644	0.479±0.013	85.332±1.298	85.331±1.298	74.436±1.973	100.000±0.000	100.000±0.000	99.998±0.000
9	0.796	0.895±0.010	94.143±0.551	94.142±0.551	100.000±0.000	99.999±0.000	88.938±0.984	100.000±0.000
10	1.003	1.019±0.019	98.927±0.667	98.927±0.667	99.710±0.407	100.000±0.000	98.171±1.527	100.000±0.000
11	1.675	1.794±0.134	94.373±2.925	94.372±2.926	97.619±1.343	99.998±0.002	91.568±6.179	99.999±0.000
12	1.966	1.497±0.043	86.438±1.407	86.434±1.407	76.140±2.177	100.000±0.000	100.000±0.000	99.992±0.001
13	2.141	3.232±0.114	79.722±1.703	79.715±1.704	100.000±0.000	99.984±0.002	66.312±2.366	100.000±0.000
14	3.749	2.228±0.242	74.359±5.634	74.349±5.635	59.444±6.457	100.000±0.000	100.000±0.000	99.977±0.004
15	4.143	3.448±0.673	85.827±4.120	85.819±4.122	78.898±10.536	99.997±0.004	95.789±5.858	99.987±0.007
16	10.108	8.351±0.327	90.453±1.946	90.440±1.948	82.621±3.236	100.000±0.000	100.000±0.000	99.975±0.005
17	12.657	10.088±0.546	88.603±2.612	88.582±2.616	79.662±4.245	100.000±0.000	99.952±0.115	99.958±0.009
18	13.063	13.492±0.246	97.656±0.899	97.652±0.901	99.254±0.000	99.992±0.004	96.124±1.730	99.999±0.000
19	15.014	11.611±0.483	87.181±2.023	87.159±2.026	77.332±3.207	100.000±0.000	99.992±0.017	99.955±0.006
20	46.828	54.497±0.726	92.426±0.663	92.374±0.668	99.990±0.000	99.894±0.010	85.933±1.146	100.000±0.000
21	56.517	59.147±0.647	97.526±0.516	97.503±0.521	99.793±0.000	99.956±0.010	95.365±0.985	99.998±0.000
22	482.939	429.534±0.915	94.147±0.106	93.675±0.114	88.942±0.189	100.000±0.000	100.000±0.000	99.056±0.016

Note: SI = similarity index; Sen. = sensitivity; Spe. = specificity; PPV = positive predictive value; NPV = negative predictive value.
